# Emotional regulation and suicidal ideation—Mediating roles of perceived social support and avoidant coping

**DOI:** 10.3389/fpsyg.2024.1377355

**Published:** 2024-04-02

**Authors:** Soham Gupta, Jonathan Fischer, Sakhi Roy, Atreyee Bhattacharyya

**Affiliations:** ^1^Amity Institute of Psychology and Allied Sciences, Amity University Kolkata, Kolkata, West Bengal, India; ^2^Department of Biostatistics, University of Florida, Gainesville, FL, United States; ^3^Amity School of Economics, Amity University Kolkata, Kolkata, India

**Keywords:** suicidal ideation, cognitive reappraisal, social support, avoidant coping, suppression

## Abstract

**Introduction:**

Recent research has uncovered a wide prevalence variation of suicidal ideation in university students ranging from 9.7% to 58.3%. India has witnessed a 4.5% increase in suicide rates in the year 2021. The interplay between cognitive reappraisal of a stressful situation, suppression of emotional expression, and coping strategies for suicidal ideation of Indian University students is yet to be explored. We aim to determine whether suicidal ideation would differ across different types of family units, and to predict the extent to which perceived social support and avoidant coping could mediate the relation between emotion regulation processes and suicidal ideation.

**Methods:**

Two hundred randomly selected University students (Mean age = 19.9, SD = 1.43) participated. Kruskal-Wallis, Pearson's product-moment correlation, and GLM mediation model were computed.

**Results and discussion:**

Lifetime suicidal ideation significantly differed between those who stay alone and those who live in a nuclear family (*p* < 0.01), and also those who stay in a joint family (*p* < 0.05). Cognitive reappraisal predicted a reduction in suicidal ideation mediated by perceived social support (B = −0.06, *p* < 0.05) and avoidant coping (B = −0.07, *p* < 0.05). Whereas, expressive suppression predicted induced levels of suicidal ideation through perceived social support (B = 0.05, *p* < 0.05), and avoidant coping (B = 0.06, *p* < 0.05) as mediators.

**Conclusion:**

Though our sample size restricts the generalization, our findings implied the importance of regular psychological consultation regarding the efficacy of the said coping processes in dealing with suicidal ideation.

## Introduction

In recent years, the alarming increase in suicide rates has become a pressing public health concern, casting a dark shadow over the wellbeing of societies worldwide (World Health Organization, 2020). Particularly, within the educational landscape, suicide has emerged as a poignant issue, with its prevalence soaring among young individuals, making it the leading cause of death among youths in India (Vijayakumar et al., 2019). A recently published meta-analysis showed a wide range (9.7% to 58.3%) of suicidal ideation rates among university students (Crispim et al., 2021). The latest data from the National Crime Records Bureau underscores this grim reality, revealing a 4.5% increase in suicide rates in the year 2021 (Verma, 2022) and has revealed that the second highest number of suicides (32.8%) in India is between 18 to 30 years of age. Suicide is an intentional effort causing one's own death with an awareness of the probable consequence (Lee et al., 2008; Song and Bae, 2020). Suicidal ideation is a broad term often involving a sense of contemplation and preoccupation with death and especially with suicide (Harmer et al., 2024). Suicidal ideation ranges from abstract thoughts about death (passive ideation) to having a specific (active ideation) suicidal plan (Song and Bae, 2020).

The transition to college and university demands significant change in the sense of independence, social demands and academic challenges amidst reduced levels of parental support (Holdaway et al., 2018), resulting in the exacerbation of psychological complexities (Akram et al., 2020; Karyotaki et al., 2020). The classic interpersonal theory of suicide (Joiner, 2005; Van Orden et al., 2010; Carpenter et al., 2015) poses much relevance in this context. It is the simultaneous presence of the two interpersonal constructs, namely, thwarted belongingness (sense of loneliness, having fewer friends) and perceived burdensomeness (a sense of liability to the parents) at this transitional phase that contributes to suicidal desires. The presence of either the thwarted sense of belongingness or burdensomeness can induce passive suicidal ideation; whereas, in the case of the presence of both factors, active suicidal ideation can be reinforced and may even lead to suicidal behavior (Song and Bae, 2020). The need for acceptance and recognition by their peers, perceived turmoil in interpersonal relationships, and fear of negative evaluations are often found to be valid predictors of suicidal ideation (Preston et al., 2022). Due to such fears and concerns, students often start to neglect social interaction, resulting in restricted social and psychological functioning, and even suicidal thoughts (Preston et al., 2022). Previous studies put much emphasis on the suicidal ideation of university students (Bernanke et al., 2017; Akram et al., 2020; Tasnim et al., 2020; Pillay, 2021) uncovering psychological problems (Cvetkovski et al., 2017) and especially explored possible factors associated with the risk of suicide, including depression (contributing 47% to 74% of risk), substance abuse, eating disorders, and schizophrenia (Yin et al., 2020; Correll et al., 2022; Favril et al., 2022). Baldini et al. (2023) in their recent meta-analysis found that the exposure to adverse childhood experiences strongly increases the probability of a suicidal attempt in individuals having schizophrenia spectrum disorders. Miola et al. (2023) further highlighted how psychiatric hospitalization, self harm, and nicotine use can independently contribute to suicidal thoughts and attempts in individuals having first episode mania or psychosis. Nonetheless, there remains a need to uncover the potential roles of adaptive and maladaptive coping processes to deal with suicidal ideation and its connection to emotional regulation.

Coping is a cognitive and behavioral tactic to reduce stress by finding ways to endure and minimize the impact of a stress-inducing situation and any associated negative emotions (Al-Dajani et al., 2022). Research has explored how coping strategies adapt in response to changes in stressors and life events (Cepuch et al., 2023). Less use of coping and problem solving initiatives often heighten the chance of suicidal ideation (Tang and Qin, 2015). Maladaptive coping strategies like disengaging behavior, passive coping, denial, substance use, self-blame, avoidant coping, and passive coping have been found to positively associate with suicidality (Chou et al., 2018; Nicoară et al., 2023). Denial is often used as an avoidant coping strategy, and studies demonstrate a positive relationship between such maladaptive coping and suicidal ideation (Horwitz et al., 2018; Werbart Törnblom et al., 2021). The perception of avoidant coping as a maladaptive one its contribution in suicidal ideation is still inconclusive. Ample studies suggest that avoidant coping is often perceived as a refuge from the problem situation, hence those individuals are liable to quit or withdraw efforts to reduce stressors (Blankstein et al., 2007; Woodhead et al., 2014); whereas others suggest avoidant coping as a temporary shift of focus to other important facets which may reduce the occurrence of suicidal thoughts (Wang et al., 2007; Miotto and Preti, 2008). On the other hand, perceived social support, which has been classically considered an adaptive and healthy coping process, has been found to negatively associate with avoidant coping and positively associate with adaptive coping strategies among college students (Calvete and Connor-Smith, 2007). In a study of Chinese college students, Cheng et al. (2020) found perceived social support from family members to be a stronger predictor of lower levels of suicidal ideation than perceived social support from friends. College students displaying higher levels of perceived social support showed better emotion regulation abilities along with reduced anxiety and depressive symptoms (Shi, 2021). Further, Perceived parental support was found to associate with lower levels of suicidal ideation among adolescents from nuclear families (Takizawa et al., 2017).

The ability to modify an individual's appraisal of a situation, emotional state, or the emotional significance of a situation establishes cognitive reappraisal as an effective emotion regulation strategy (Gross, 2014). Emotion regulation is often conceptualized as experiencing the control over emotional experience and expression (Gratz and Roemer, 2004). An adaptive emotion regulation involves modulating the intensity and magnitude of an emotional experience rather than inhibiting inappropriate ones (Thompson and Calkins, 1996; Gratz and Roemer, 2004). Difficulties in regulation of emotion were associated with a range of mental health problems, including depression, anxiety, and borderline personality disorder (Berking et al., 2019). Cognitive reappraisal, having the ability to modify an individual's appraisal of a situation, has been found to moderate the relationship between perceived social support and suicidal ideation. A recent meta-analysis on the role of emotion regulation on suicidal ideation found mindfulness and cognitive appraisal to be adaptive strategies to regulate emotion and suppression of emotional expression; avoidance and broadening were considered to be maladaptive emotional regulation processes (Rogier et al., 2024).

Family structure is often found to be associated with the propensity to experience suicidal ideation (Jadav, 2020). Ahookhosh et al. (2017) found that individuals from nuclear families had a lower risk of suicidal ideation compared to those from joint families. Another study found that individuals living alone had a higher risk of suicidal ideation compared to those living in nuclear or joint families (Olfson et al., 2022). In a study of Chinese university students, Chu et al. (2021) found that higher levels of perceived social support were associated with lower levels of suicidal ideation with stress as the mediator. Modernity-driven changes in family structure, perception of being neglected or ignored by parents, excessive use of social media, a history of childhood trauma were often identified as suicide related risk factors (Kar, 2010; Bhosle et al., 2015; Senapati et al., 2024) in India.

Previous work has explored the interplay between emotional regulation, coping processes, and suicidal ideation (Jose and Angelina, 2020; Wastler and Núñez, 2022). A recently published article reviews and explains how the inability to cope with daily life stressors, the absence of peer group or family support, and not having kin around often have a debilitating effect on suicidal ideation (Senapati et al., 2024). At this standpoint our study aims to contribute to the existing literature by exploring the extent to which cognitive reappraisal and expressive suppression predict suicidal ideation while considering perceived social support and avoidant coping as potential mediating factors. Our results explore the extent to which these studied coping processes can be found responsible in inducing or reducing suicidal ideation. To the best of our knowledge, this is the first study on university students in the Indian subcontinent exploring the associations of coping strategies with the aforementioned variables.

Our study's objectives are:

i) To assess the extent to which suicidal ideation differs across different types of family units;
ii) To quantify and describe any associations among coping style, emotional regulation, perceived social support, and suicidal ideation; and
iii) To explore the extent to which perceived social support and avoidant coping act as mediators of the associations between cognitive reappraisal and suicidal ideation as well as between expressive suppression of emotion and suicidal ideation.

**Figure 1:**
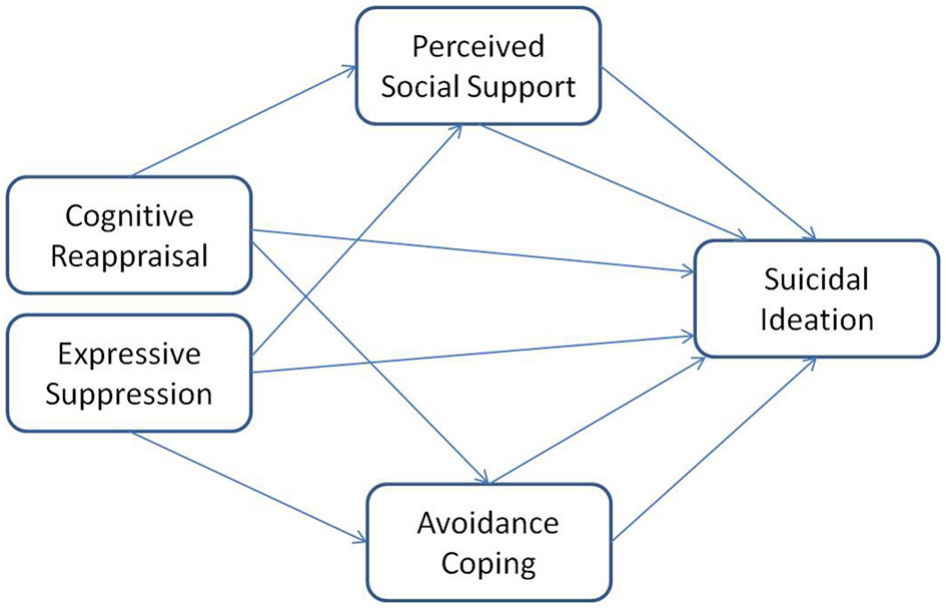
The conceptual framework exploring the potential predictive variables,—cognitive reappraisal and expressive suppression predicting suicidal ideation through perceived social support and avoidant coping as mediators.

The conceptual framework of the interplay between the variables is depicted in Figure 1.

## Materials and methods

### Participants and procedure

A cohort of 200 participants underwent assessment subsequent to providing informed consent for the collection, processing, and publication of anonymized data. Respondents exclusively comprised students enrolled in either undergraduate or post-graduate programs at universities in Kolkata (Mean age = 19.9 and SD = 1.43). Participants were randomly sampled to ensure a representative and unbiased sample from the target population. Individuals with a documented history of neurotic and psychotic spectrum disorder diagnosed by a certified clinical psychologist or a psychiatrist were intentionally excluded from the administration of the questionnaires. Explicit informed consent was obtained from all participants before the direct administration of the aforementioned scales. Data acquisition transpired in an offline mode, facilitated by the distribution of hard-copy questionnaires. All procedures performed in studies involving human participants were in accordance with the ethical standards of the institutional research committee [DRC-AIPSK/ETHICS/A91316621022] and with the 1964 Helsinki Declaration and its later amendments or comparable ethical standards.

### Measures

#### Brief-COPE

The Brief-COPE, a concise 28-item scale, measures coping strategies via a 4-point Likert scale. It is a condensed version of the initial 60-item COPE scale that was theoretically constructed based on different coping mechanisms (Carver, 1997). It assesses problem-focused coping (“I take action”), emotion-focused coping (“I focus on something else”), and avoidant coping (“I avoid reminders”). Higher scores denote increased usage of respective strategies. In this study, subscale Cronbach's alpha values are 0.73 for problem-focused, 0.61 for emotion-focused, and 0.68 for avoidant coping. The total coping score yields a Cronbach's alpha of 0.75. These values signify moderate to acceptable internal consistency. The instrument efficiently captures diverse coping mechanisms in stressful situations.

#### Emotion regulation questionnaire

The Emotion Regulation Questionnaire (ERQ) devised by Gross and John (2003), gauges cognitive reappraisal and expressive suppression strategies through 10 items on a 7-point Likert scale. Cognitive reappraisal items (1, 3, 5, 7, 8, 10) focus on altering thoughts for positive emotions (for example, “When I want to feel less negative emotion …. I change what I'm thinking about”), while expressive suppression items (2, 4, 6, 9) assess emotion concealment (for example, “I keep my emotions to myself”). Higher scores indicate greater strategy use. Among Indian college students, the ERQ exhibited high internal consistency and test-retest reliability. In the present study, cognitive reappraisal scored 0.79, expressive suppression 0.71, and ERQ total 0.74, showcasing robust reliability.

#### Multidimensional scale of perceived social support

The MSPSS, developed by Zimet et al. (1988), gauges perceptions of social support (for example, “There is a special person who is around when I am in need”) with 12 items on a 7-point Likert scale ranging from “very strongly disagree” (1) to “very strongly agree” (7). Subscales include family, friends, and significant others. Higher scores indicate greater support. Strong reliability and validity has been evidenced. In this study, Cronbach's alphas were 0.91 for family, 0.9 for friends, 0.94 for significant others, and 0.86 for the total score.

#### Suicide behaviors questionnaire revised

SBQ-R by Osman et al. (2001) comprises four items, addressing various aspects of suicidality. The Likert scale differs for all the four items. Item 1 explores past suicidal thoughts/attempts. The scale ranges from “Never” (1) to “Have attempted … hoped to die” (4b). Item 2 assesses the frequency of suicidal thoughts in the past 12 months where the scale ranges from “Never” (1) to “Very often” (5), and item 3 evaluates the threat of a suicide attempt ranging from “No” (1) to “Yes, more than once….” (3). Item 4 gauges self-reported propensity for future suicidal behavior, with a scale ranging from “Never” (0) to “Very likely” (6). The total score is the aggregate of the scores of all the four items. In this study, the SBQ-R demonstrated a reliability score of 0.8.

### Procedure

Participants were randomly chosen. All the above-mentioned scales were administered directly to the participants only after receiving informed consent. Data was collected offline through hard-copies of questionnaires with distractions minimized. Adequate rest periods were given between the administration of different tests to avoid fatigue. Doubt(s), if any, regarding the questionnaires were cleared. For the grouping, analysis, and interpretation of data, Microsoft Excel, Jamovi (version 2.3.26.0), and R were used. Statistical significance was determined at a level of 0.05.

### Analysis

The mean, median, and standard deviation were calculated for quantitative variables as descriptive statistics. To ensure robustness against observed departures from the assumptions of parametric one-way ANOVA, a non-parametric alternative, the Kruskal-Wallis test, was employed to test for an association between different family unit types and the variables defined in **Table 2**. To investigate the relationships among variables, Pearson's product moment correlation was computed. Given the nature of these variables, Spearman's rank correlation was also computed and yielded nearly identical results. Finally, a mediation model was used to determine if social support and avoidant coping could mediate the association of cognitive reappraisal and expressive suppression with suicidal ideation, and further, if mediated, whether the mediation was complete or partial. The mediation model results were robust to various choices of variable transformation or response distribution for possibly right-skewed data; the article presents results using the standard linear model formulation.

## Results

**Figure 2:**
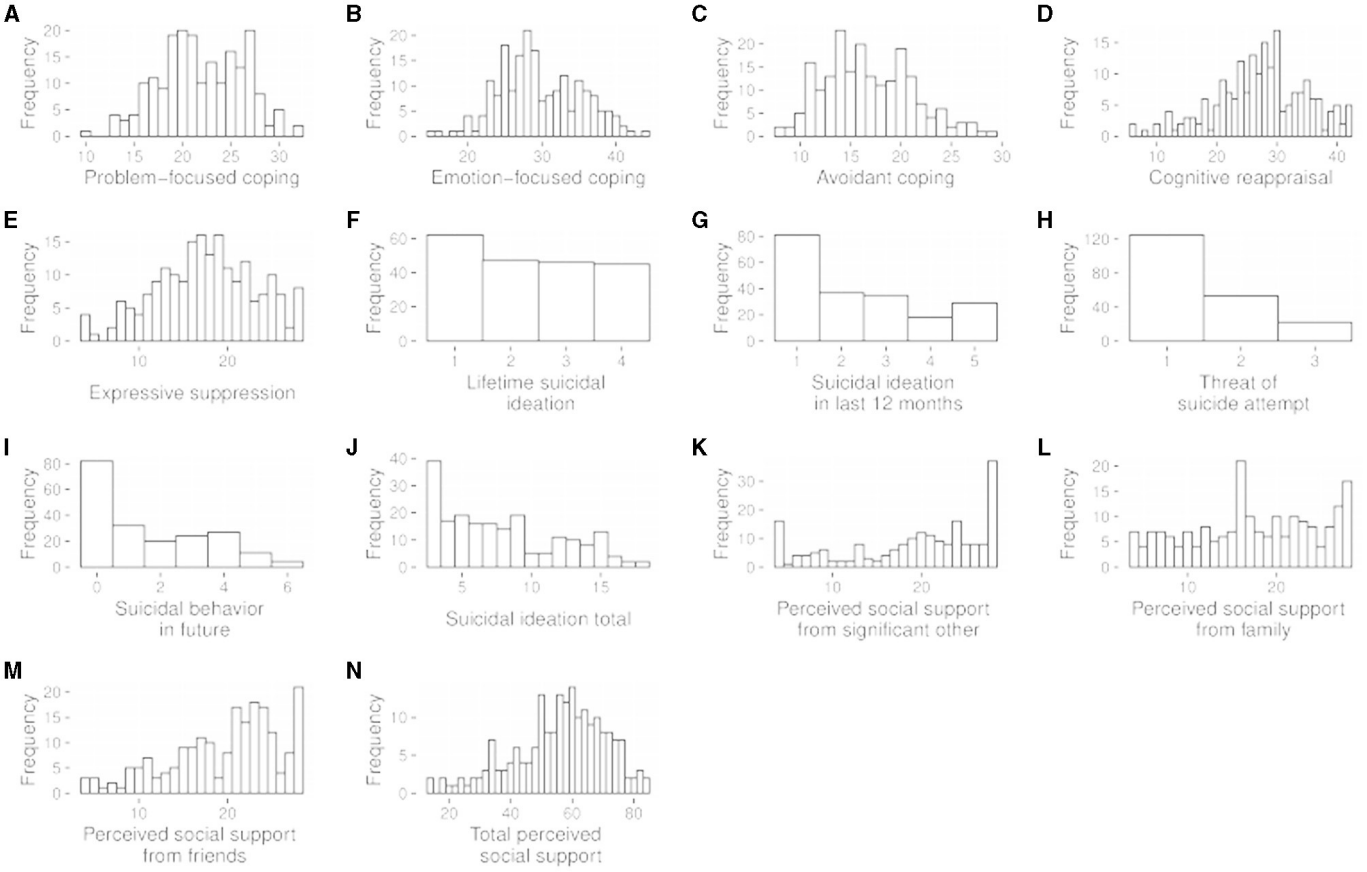
Histograms showing distribution of response score values (*N* = 200 for each quantity). Selected numerical summaries of these distributions are presented in Table 1. **(A)** Histograms showing distribution of response score of Problem-focused coping. **(B)** Histograms showing distribution of response score of Emotion-focused coping. **(C)** Histograms showing distribution of response score of Avoidant coping. **(D)** Histograms showing distribution of response score of Cognitive reappraisal. **(E)** Histograms showing distribution of response score of Expressive suppression. **(F)** Histograms showing distribution of response score of Lifetime suicidal ideation. **(G)** Histograms showing distribution of response score of Suicidal ideation in last 12 months. **(H)** Histograms showing distribution of response score of Threat of suicide attempt. **(I)** Histograms showing distribution of response score of Suicidal behavior in future. **(J)** Histograms showing distribution of response score of Suicidal ideation total. **(K)** Histograms showing distribution of response score of Perceived social support from significant other. **(L)** Histograms showing distribution of response score of Perceived social support from family. **(M)** Histograms showing distribution of response score of Perceived social support friends. **(N)** Histograms showing distribution of response score of Total perceived social support.

Table 1 reports the percentile values for each of the measured variables along with their standard deviations and Shapiro-Wilk test results; most variables displayed a lack of normality. Figure 2 represents histograms showing distribution of response score values (*N* = 200 for each quantity). Selected numerical summaries of these distributions are presented in Table 1.

**Table 1:** Interquartile range, median, standard deviation (SD) of all the variables (*N* = 200).

		**Shapiro-Wilk**		**Percentiles**		
	**SD**	**W**	**P**	**25th**	**50th**	**75** ^th^
PF	4.321	0.983	0.017	19.00	21.50	25.25
EF	5.488	0.984	0.023	25.75	29.00	34.00
AC	4.387	0.976	0.002	14.00	16.00	20.00
Coping Total	10.056	0.990	0.155	61.00	68.00	75.00
CR	7.594	0.984	0.024	23.00	28.00	33.00
ES	5.631	0.984	0.023	14.00	18.00	22.00
ER Total	10.247	0.984	0.024	38.00	44.50	53.00
Lifetime SI	1.144	0.844	< 0.001	1.00	2.00	3.00
SI in past 12 months	1.452	0.820	< 0.001	1.00	2.00	3.00
Threat of suicide attempt	0.687	0.685	< 0.001	1.00	1.00	2.00
S B in future	1.781	0.833	< 0.001	0.00	1.00	3.00
Suicidal Ideation Total	4.223	0.909	< 0.001	4.00	7.00	11.25
S O	7.747	0.890	< 0.001	13.75	21.00	26.00
Family	7.168	0.951	< 0.001	12.00	17.00	23.00
Friends	6.157	0.940	< 0.001	16.00	21.00	24.00
SS Total	14.972	0.970	< 0.001	49.00	58.00	67.00

PF, Problem Focused coping; EF, Emotion Focused coping; AC, Avoidant coping; CR, Cognitive Reappraisal; ES, Expressive Suppression; ER Total, Emotional Regulation total score; Lifetime SI, Lifetime Suicidal ideation; SI in past 12 months, Suicidal Ideation in past 12 months; SB in future, Suicidal Behavior in future; S O, Social support by Significant Others; SS Total, Social Support Total score.

### Exploring mean difference of suicidal ideation across different types of family units

For the verification of our first objective, non-parametric one-way ANOVA was conducted (Table 2). The results demonstrate that the type of family unit a student belongs to has a significant association with lifetime suicidal ideation and/or attempt ($\chi_{2}^{2}$ = 7.169, *p* < 0.05). Visual examination of the distribution of lifetime suicidal ideation and/or attempt scores across family unit categories indicated the significant result was being driven by the living alone group having higher scores. Indeed, *post-hoc* pairwise testing (Table 3) showed a significant difference between this group and both the Nuclear family ($\chi_{1}^{2}$ = 6.850, *p* = 0.009) and Joint ($\chi_{1}^{2}$ = 4.163, *p* = 0.041) groups, but no difference between the Nuclear family and Joint groups ($\chi_{1}^{2}$ = 0.06, *p* = 0.806); all results were computed via Kruskal-Wallis tests. Other variables did not display significant associations with family unit type. Despite varying levels of statistical significance, for all four considered response variables we provide the mean difference in score across each pairwise comparison of family unit type. These are accompanied by 95% confidence intervals obtained via bootstrapping in order to allow a more holistic assessment of the strength and precision of our observed associations.

**Table 2:** One-way non-parametric ANOVA (Kruskal-Wallis).

**Dependent variable**	**χ^2^**	**df**	***P***	**Alone vs. nuclear**	**Alone vs. joint**	**Joint vs. nuclear**
Lifetime suicidal ideation and/or attempt	7.169	2	0.028^*^	0.57 (0.15, 0.98)	0.54 (0.02, 1.03)	0.04 (−0.36, 0.43)
Threat of suicide attempt	2.419	2	0.298	0.13 (−0.12, 0.39)	−0.07 (−0.40, 0.27)	0.20 (−0.07, 0.48)
Likelihood of suicidal behavior in future	1.119	2	0.572	0.37 (−0.33, 1.07)	0.46 (−0.39, 1.29)	−0.09 (−0.73, 0.57)
Suicidal Ideation Combined Total	2.975	2	0.226	1.35 (−0.22, 2.93)	1.48 (−0.49, 3.42)	−0.14 (−1.65, 1.40)

^*^p < 0.05.

**Table 3:** *Post-hoc* tests: lifetime suicidal ideation/attempt vs. family unit.

**Family unit comparison**	**χ^2^**	**df**	***P***
Living alone vs. nuclear	6.85	1	0.009^*^
Living alone vs. joint	4.16	1	0.041^*^
Nuclear vs. joint	0.06	1	0.806

^*^p < 0.05.

**Figure 3:**
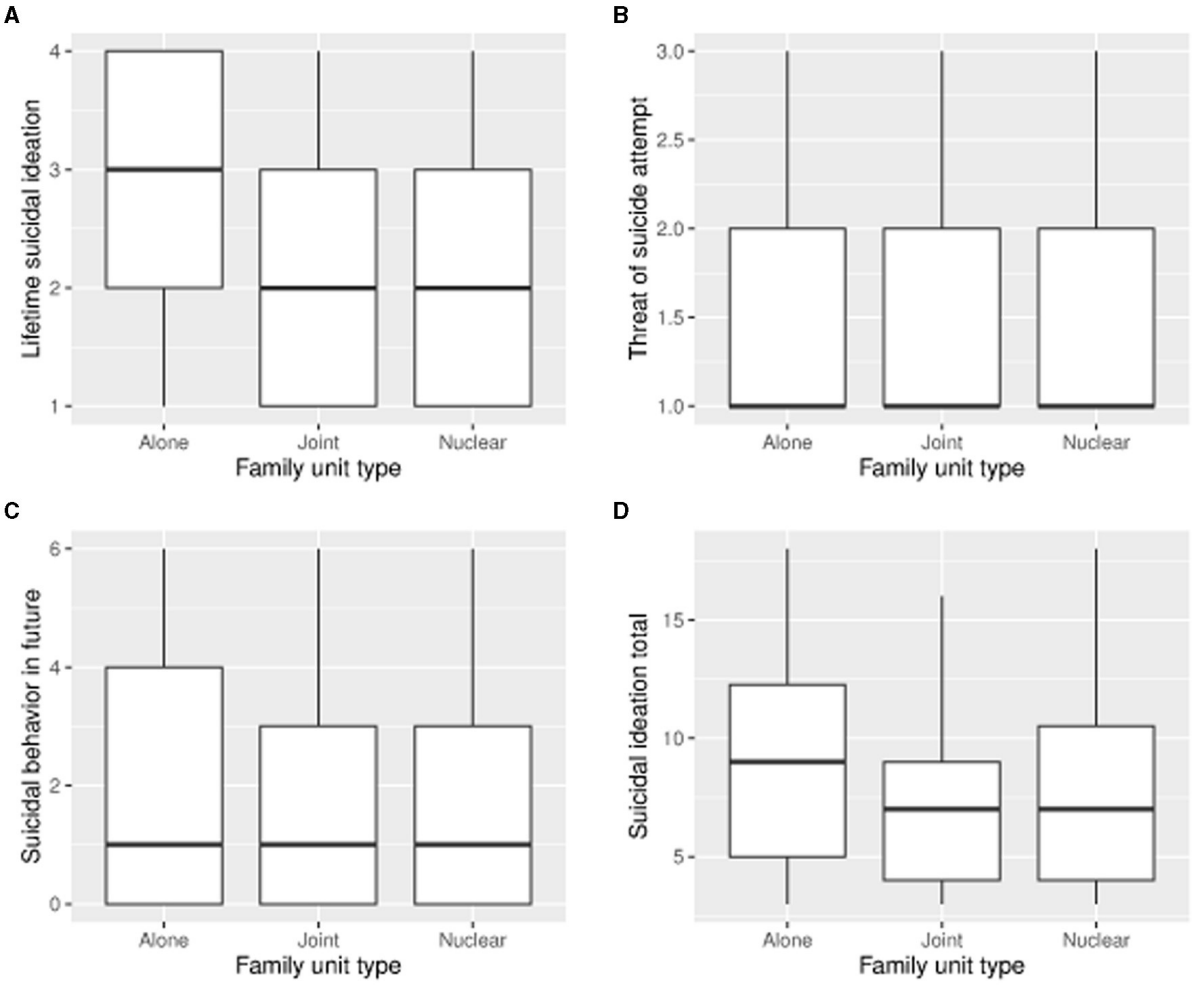
Box plots of selected response scores as a function of family unit type (*N* = 36 for alone, *N* = 37 for Joint, *N* = 127 for Nuclear). **(A)** Box plots of Lifetime suicidal ideation scores as a function of family unit type. **(B)** Box plots of Threat of suicide attempt scores as a function of family unit type. **(C)** Box plots of Suicidal behavior in future scores as a function of family unit type. **(D)** Box plots of Suicidal ideation total scores as a function of family unit type.

Figure 3 presents box plots of selected response scores as a function of family unit type.

### Relationships between the stated variables

**Figure 4:**
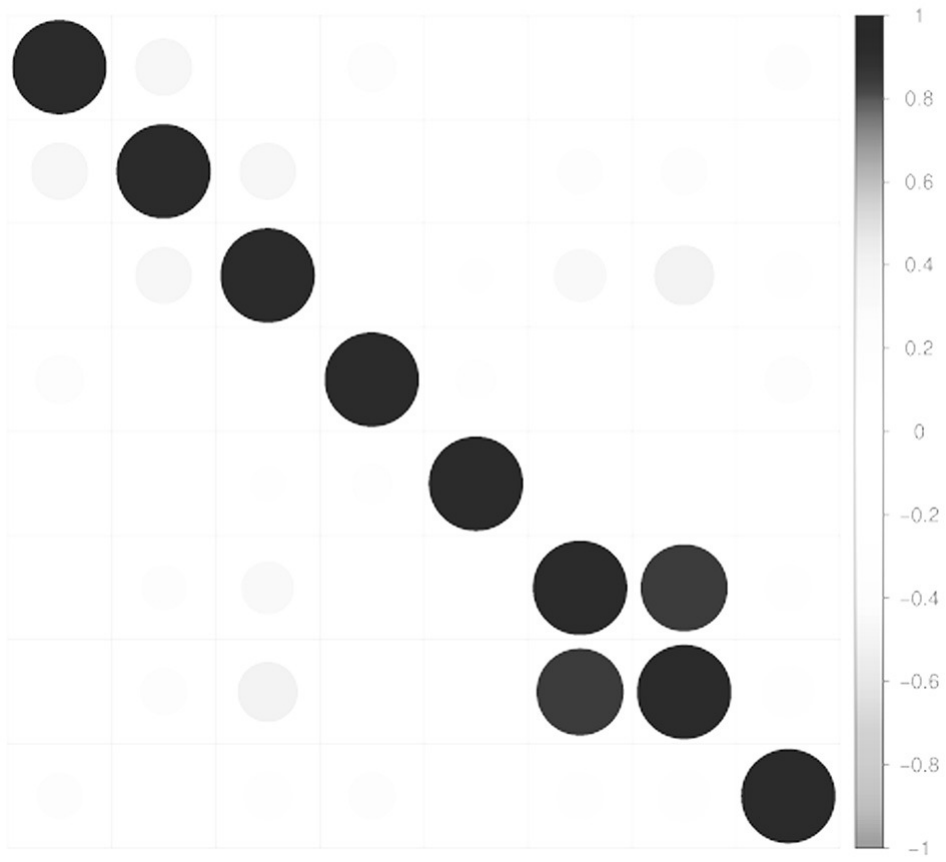
Correlation plot of the Pearson correlation for each variable pair. For each pair, the circle size and color match the Pearson correlation value as indicated by the color bar on the right side of the figure.

To quantify and test the significance of measured relationships between variables, the Pearson Product-Moment correlation was computed for each variable pair. Significant positive correlations were identified between problem-focused coping and number of variables, including cognitive reappraisal (r = 0.264, *p* < 0.001), total social support score (r = 0.23, *p* < 0.001), and emotion-focused coping (r = 0.37, *p* < 0.001). Emotion-focused coping further demonstrated noteworthy positive relationships with avoidant coping (r = 0.363, *p* < 0.001), lifetime suicidal ideation (r = 0.224, *p* < 0.001), and total suicidal ideation score (r = 0.243, *p* < 0.001). Lastly, avoidant coping displays significant negative associations with cognitive reappraisal (r = −0.185, *p* < 0.01) and social support total score (r = −0.283, *p* < 0.001), but significant positive associations with expressive suppression (r = 0.147, *p* < 0.05), lifetime suicidal ideation (r = 0.314, *p* < 0.001), and suicidal ideation total score (r = 0.405, *p* < 0.001). Figure 4 displays a correlation plot for variable pairs, and the full set of correlation values and *p*-value are shown in Table 4.

**Table 4:** Pearson's product-moment correlation coefficients and corresponding *p*-values.

		**PF**	**EF**	**AC**	**CR**	**ES**	**Lifetime SI**	**SIT**	**SS Total**
PF	Pearson's r	—							
	*p*-value	—							
EF	Pearson's r	0.370	—						
	*p*-value	< 0.001	—						
AC	Pearson's r	−0.05	0.363	—					
	*p*-value	0.478	< 0.001	—					
CR	Pearson's r	0.264	−0.06	−0.185	—				
	p-value	< 0.001	0.391	0.01^**^	—				
ES	Pearson's r	−0.09	−0.03	0.147	0.183	—			
	*p*-value	0.209	0.677	0.038^*^	0.010^**^	—		
Lifetime SI	Pearson's r	−0.054	0.224	0.314	−0.167	0.079	—		
	*p*-value	0.449	0.001	< 0.001	0.018^*^	0.266	—		
SIT	Pearson's r	−0.062	0.243	0.405	−0.200	0.102	0.848	—	
	*p*-value	0.381	< 0.001	< 0.001	0.004^**^	0.150	< 0.001	—	
SS Total	Pearson's r	0.230	0.071	−0.283	0.244	−0.193	−0.271	−0.323	—
	*p*-value	0.001	0.319	< 0.001	< 0.001	0.006^**^	< 0.001	< 0.001	—

PF, Problem Focused coping; EF, Emotion Focused coping; AC, Avoidant coping; CR, Cognitive Reappraisal; ES, Expressive Suppression; Lifetime SI, Lifetime Suicidal ideation; SIT, Suicidal Ideation total score; SS Total, Social Support Total score. ^*^p < 0.05, ^**^p < 0.01.

Figure 4 is a correlation plot of the Pearson correlation for each variable pair, visualizes the correlation matrix provided in Table 4.

### Application of the mediation model

**Figure 5:**
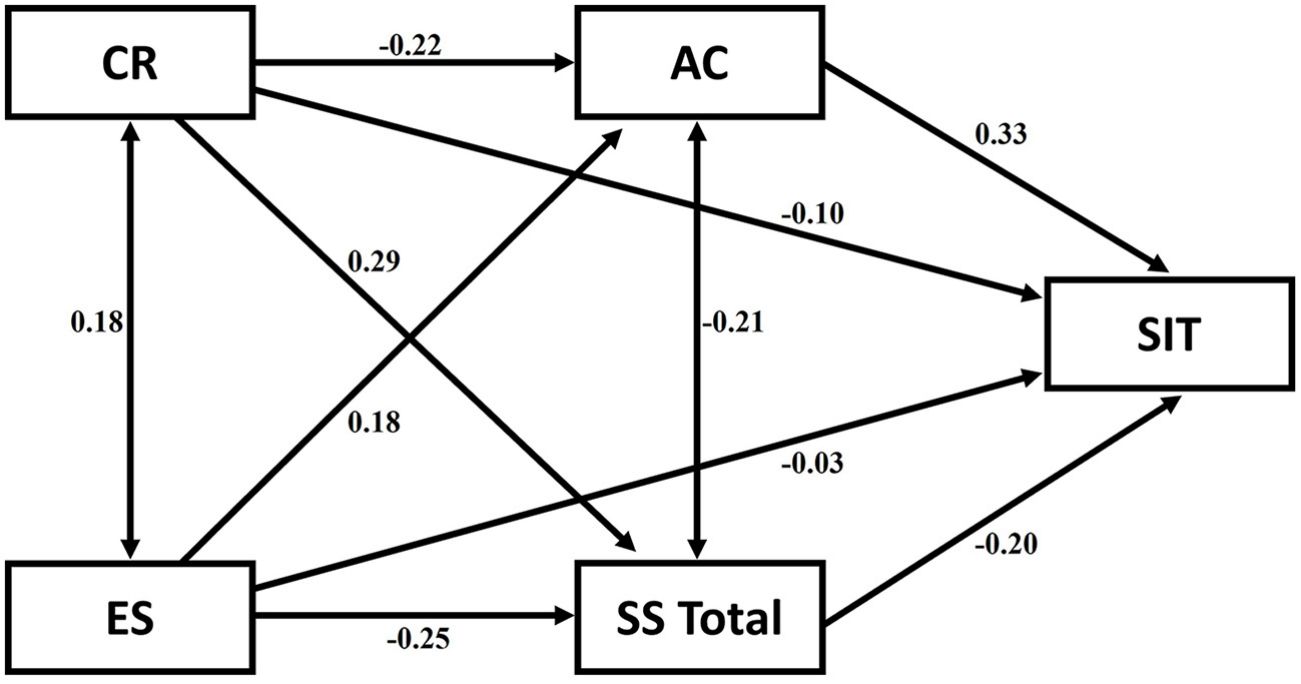
Mediation pathways between cognitive reappraisal (CR), expressive suppression (ES), perceived social support (SS Total), avoidant coping (AC), and suicidal ideation (SIT).

To deconstruct the observed set of correlations with total suicidal ideation and explore the possibility of mediation by avoidant coping and total social support, both mediation and path estimates (Table 5) were calculated according to the model depicted in Figure 5.

**Table 5:** Indirect and total associations.

				**95% C.I. (a)**				
**Type**	**Effect**	**Estimate**	**SE**	**Lower**	**Upper**	**B**	**Z**	**P**
Indirect	ES ⇒ SS Total ⇒ SIT	0.0371	0.016	0.00968	0.0728	0.0494	2.298	0.022^*^
	ES ⇒ AC ⇒ SIT	0.0456	0.020	0.01466	0.0944	0.0607	2.282	0.022^*^
	CR ⇒ SS Total ⇒ SIT	−0.0324	0.015	−0.06854	−0.0109	−0.0583	−2.202	0.028^*^
	CR ⇒ AC ⇒ SIT	−0.0396	0.016	−0.07831	−0.0139	−0.0712	−2.520	0.012^*^
Component	ES ⇒ SS Total	−0.6526	0.181	−0.96332	−0.2624	−0.2454	−3.614	< 0.001
	SS Total ⇒ SIT	−0.0568	0.019	−0.09696	−0.0171	−0.2014	−2.911	0.004^**^
	ES ⇒ AC	0.1456	0.055	0.04155	0.2518	0.1869	2.619	0.009^**^
	AC ⇒ SIT	0.3129	0.058	0.20458	0.4304	0.3251	5.336	< 0.001
	CR ⇒ SS Total	0.5704	0.149	0.27221	0.8562	0.2893	3.809	< 0.001
	CR ⇒ AC	−0.1266	0.042	−0.20471	−0.0375	−0.2192	−2.979	0.003^**^
Direct	ES ⇒ SIT	0.0251	0.053	−0.08305	0.1255	0.0334	0.470	0.638
	CR ⇒ SIT	−0.0539	0.039	−0.13736	0.0143	−0.0970	−1.379	0.168
Total	ES ⇒ SIT	0.1077	0.052	0.00493	0.2104	0.1436	2.054	0.040^*^
	CR ⇒ SIT	−0.1259	0.038	−0.20213	−0.0498	−0.2265	−3.240	0.001

Confidence intervals computed with bias-corrected bootstrap. AC, Avoidant coping; CR, Cognitive Reappraisal; ES, Expressive Suppression; SIT, Suicidal Ideation total score; SS Total, Social Support Total score. Betas are completely standardized effect sizes. ^*^p < 0.05, ^**^p < 0.01.

Specifically, we applied mediation analysis to assess the role of Perceived Social Support (SS Total) in the connection between Cognitive Reappraisal (CR) and Suicidal Ideation (SIT), as well as between Expressive Suppression (ES) and SIT. Results (refer to Table 4) indicated significant total effects of ES (Estimate = 0.12, ß = 0.14, t = 2.05, *p* < 0.05) and CR (Estimate = −0.13, ß = −0.23, t = −3.24, *p* = 0.001) on SIT. However, upon introducing SS Total as a mediating variable, the direct effects of ES and CR on SIT became non-significant. Notably, the indirect associations through SS Total were significant for both ES (Estimate = 0.037, ß = 0.049, t = 2.29, *p* < 0.05) and CR (Estimate = −0.032, ß = −0.058, t = −2.2, *p* < 0.05), indicating full mediation of the relationship between ES and CR on SIT by SS Total.

With the inclusion of AC as a mediating variable, the direct associations between ES and SIT and between CR and SIT were not statistically significant. The indirect association between ES and SIT through the mediating variable AC was found to be significant (estimate = 0.045, ß = 0.06, *t* = 2.282, *p* < 0.05). Similarly, the indirect association between CR and SIT through the mediating variable AC was also found to be significant (Estimate = −0.039, ß = −0.071, t = −2.520, *p* < 0.05). These results support the notion that AC fully mediates the relationships between ES and SIT as well as between CR and SIT. Table 4 shows the full set of mediation estimates between cognitive reappraisal (CR), expressive suppression (ES), perceived social support (SS Total), avoidant coping (AC), and suicidal ideation (SIT).

## Discussion

Our study throws lights on the probable association between the types of family units and suicidal thoughts, and especially explored the contributions of adaptive and maladaptive coping styles mediating the relationship between emotion regulation strategies and suicidal ideation. Our study revealed a significant difference in suicidal thoughts among students in different household types, aligning with previous findings (Freudenstein et al., 2011; Paashaus et al., 2019) associating family dynamics with suicidal ideation. Parental rejection, parental low care levels, and living away from parents have been identified as risk factors for having self-destructive thoughts (Cruz et al., 2013; Donath et al., 2014; Yang et al., 2022). The positive association between living alone and incidence of suicidal thoughts was echoed in our results, as *post-hoc* testing indicated that this living status displayed higher scores in this metric than did the other two household types. A study done by Shaw et al. (2021) yielded similar results, showing that, living alone was one of the risk factors associated with suicidal ideation among students. According to Andriessen et al. (2019), social isolation, including living alone, is a risk factor for suicidal ideation among students. Our result showed some similarity with these findings showing a risk for college students who live outside their own family even if it had been a nuclear one.

Significant correlations were observed between avoidance coping and increased suicide risk and between cognitive reappraisal and reduced suicidal risk, consistent with existing literature (Ong and Thompson, 2019). Avoidance of a difficult situation only delays the inevitable, and even aggravates the situation. It does not address the problem and acts as a temporary respite. Increased emotional suppression is significantly related to lower levels of social support and social wellbeing, which is also consistent with recent findings (Chervonsky and Hunt, 2017). Decreased parental support was found to be significantly related to suicidal ideation and attempts. A study by Miller et al. (2015) reported that likelihood of suicidal attempts was higher to those who felt that friends provided very little assistance and backing. Our study supported this finding as well. Research suggests that individuals who are better able to use cognitive reappraisal are less likely to experience suicidal ideation, as they are better able to cope with stress and negative emotions by using their brain power to see things from a different perspective and deal with them in newer and innovative ways (Franz et al., 2021). This is also aligned with our study.

Our study explores how coping processes mediate the observed relationships between emotional regulation strategies and suicidal ideation. Perceived social support significantly mediated the link between expressive suppression and suicidal ideation, and our results support full mediation. Moreover, the association between cognitive reappraisal and reduced suicidal risk was found significant only when perceived social support was included as a mediating variable. Our finding corroborates the findings of Sachs-Ericsson et al. (2021). We further found that cognitive reappraisal could predict reducing levels of suicidal ideation even when the pathway involved avoidant coping as the mediator. Coping strategies related to avoidance, such as behavioral disengagement and self-destruction were found to be more likely adapted by individuals having suicidal ideation (Liang et al., 2020) reinforcing its maladaptive nature.

The association between cognitive reappraisal or expressive suppression of emotion and suicidal ideation has already been evidenced (Ong and Thompson, 2019; Turton et al., 2022). In our study, component pathways indicated that higher expressive suppression correlated with reduced perceptions of receiving social support, and that reduced perceptions of social support in turn associated with higher levels of suicidal ideation. Being a response-focused form of emotional regulation, expressive suppression inhibits the ongoing emotion-expressive behavior (Gross and John, 2003; Cutuli, 2014) which has been evidenced to associate with reduced perception of social support. Thus, in our study, higher expressive suppression indicated increased suicidal ideation via a reduction in perceived social support.

On the other hand, by modifying or altering an individual's appraisal of a situation or emotional state, cognitive appraisal has often been found to associate with lowered suicidal risk, and studies have demonstrated the role of cognitive reappraisal in moderating the effect of depressive symptoms on perceiving social support (Ong and Thompson, 2019; Sachs-Ericsson et al., 2021). Owen et al. (2021) showed a significant meditational pathway between perceived social support and changes in suicidal ideation over time through making changes in the sense of defeat, entrapment, and hopelessness.

Individuals employing avoidant coping show a tendency to refrain from confronting problem situations and addressing stressors, often correlate with a higher risk of suicidality (Liang et al., 2020). Strategies like substance use, denial, and behavioral as well as mental disengagements are avoidant coping strategies of a maladaptive type (Ong and Thompson, 2019), whereas active coping processes like perceived social support involve attempts to resolve stressors. Our study revealed that suppression of emotional and behavioral expression positively correlate with avoidant coping, which is in turn associated with higher levels of suicidal ideation. Conversely, cognitive reappraisal, recognizing the probable harm that the stressor could inflict, negatively correlates with avoidant coping, thus associating with reduced levels of suicidal ideation. The significant associations and mediating roles for perceived social support and avoidant coping found in our analyses emphasize the importance of adaptive coping strategies and their potential to amplify or diminish the effect of stressors.

Future directions in research on suicidal ideation and prevention may shift toward personalized interventions, capitalizing on precision in medicinal advancements and a deeper understanding of individual risk factors (World Health Organization., 2023). Advancements in neuroscience and psychological research could lead to the identification of biomarkers associated with suicide risk. Integrating these biomarkers into routine assessments may facilitate early identification and targeted interventions for individuals at risk. The integration of technology and digital mental health tools is poised to play a crucial role. Mobile applications, wearable devices, and online platforms could enable early detection, continuous monitoring, timely intervention, and the establishment of emergency telemedicine services (Accorsi et al., 2023). Machine learning algorithms and predictive analytics may enhance risk assessment models by analyzing vast data sets, such as biological markers, behavioral patterns, and social interactions (Boudreaux et al., 2021).

A central focus is anticipated to be the strengthening of community-based programs and public health initiatives. Increasing awareness, reducing stigma, and fostering supportive environments are critical components (Khoury et al., 2015; Government of India, 2019). Recognizing the significance of early identification and intervention, future efforts might integrate mental health education and support within educational institutions, workplaces, and primary care settings. Our findings pose much significance here. Intervention should concentrate on acknowledging social support so as to feel less inhibited to seek support from family. In-depth evaluation before adapting coping processes, such as the avoidance of stress inducing situations, is a major implication of our study. The National Suicide Prevention Strategy of the Government of India offers valuable insights globally, emphasizing culturally sensitive approaches (Ransing et al., 2023; Rogier et al., 2024). Acknowledging diverse cultural nuances surrounding mental health and suicide, the strategy underscores the importance of community engagement, and mental health education. It encourages data-driven approaches, policy integration, and collaborative research efforts to comprehensively address suicide risk. Suicide prevention efforts might benefit from increased global collaborations and data sharing. Learning from diverse cultural perspectives and pooling data from various regions could enhance our understanding of risk factors and the effectiveness of prevention strategies (World Health Organization, 2020; Ransing et al., 2023).

## Conclusion

The analyses of our three defined objectives yielded statistically significant findings, offering valuable insights into the complex dynamics of suicidal ideation among students. Firstly, the study revealed a compelling association between the type of family unit and suicidal ideation, emphasizing a higher prevalence among students living alone. This highlights the need for targeted interventions and support systems for individuals in such family structures.

Secondly, the intricate interplay among coping styles, emotion regulation, perceived social support, and suicidal ideation underscores the multifaceted nature of mental health challenges faced by students. Identifying these nuanced connections provides a basis for developing comprehensive mental health programs tailored to the diverse needs of the student population.

Moreover, the observation of complete mediation between cognitive reappraisal and suicidal ideation by social support signals the potential efficacy of targeted interventions focusing on enhancing social support networks. Strengthening these networks could be a crucial component of mental health strategies in academic institutions.

Despite the robust methodology employed with our randomly selected sample of 200 participants, acknowledging certain limitations is crucial. While the sample was meticulously chosen, a larger and more diverse participant pool would contribute to the robustness and generalizability of our findings. Additionally, recognizing staying with a single parent as a distinct family structure and exploring its impact on suicidal ideation could further enrich our understanding of these complex dynamics.

Furthermore, the cross-sectional, correlational design used in our study, while illuminating associations, limits our ability to establish causal relationships. Future research endeavors should consider employing longitudinal or experimental designs to unravel the temporal aspects and causal mechanisms involved in suicidal ideation among students.

In conclusion, our study not only highlights the urgency for mental health resources and support in Indian universities but also suggests potential avenues for targeted interventions. Emphasizing the role of family structures, understanding intricate psychological processes, and recognizing the mediating impact of social support are critical steps toward developing comprehensive strategies to address the pressing issue of suicidal ideation among students.

## Data availability statement

The datasets presented in this study can be found in online repositories. The names of the repository/repositories and accession number(s) can be found below: https://osf.io/hwbdz/?view_only=5c6b7fd60ebf4ba090c483c63ce9cde7.

## Ethics statement

The studies involving humans were approved by Amity University Kolkata (DRC-AIPSK/ETHICS/A91316621022). The studies were conducted in accordance with the local legislation and institutional requirements. The participants provided their written informed consent to participate in this study.

## Author contributions

SG: Writing—original draft, Visualization, Data curation. JF: Formal analysis, Software, Writing—review & editing, Validation, Visualization. SR: Methodology, Writing—review & editing. AB: Conceptualization, Formal analysis, Methodology, Software, Supervision, Writing—original draft, Writing—review & editing.

## References

[B1] Accorsi T. A. D. De Amicis Lima K. Köhler K. F. Cordioli E. Pedrotti C. H. S. (2023). Assessment of suicidal ideation via telemedicine: a case report and management suggestions. Int. J. Emer. Med. 16:84. 10.1186/s12245-023-00557-2 37953263 PMC10641932

[B2] Ahookhosh P. Bahmani B. Asgari A. Moghaddam H. H. (2017). Family relationships and suicide ideation: the mediating roles of anxiety, hopelessness, and depression in adolescents. Int. J. High Risk Behav. Addict. 6:e31573. 10.5812/ijhrba.31573

[B3] Akram U. Ypsilanti A. Gardani M. Irvine K. Allen S. Akram A.. (2020). Prevalence and psychiatric correlates of suicidal ideation in UK university students. J. Affect. Disor. 272, 191–197. 10.1016/j.jad.2020.03.185 32379615

[B4] Al-Dajani N. Horwitz A. G. Czyz E. K. (2022). Does coping reduce suicidal urges in everyday life? Evidence from a daily diary study of adolescent inpatients. Depr. Anxiety 39, 496–503. 10.1002/da.23253 35322919 PMC9246857

[B5] Andriessen K. Krysinska K. Kõlves K. Reavley N. (2019). Suicide postvention service models and guidelines 2014–2019: a systematic review. Front. Psychol. 10:491007. 10.3389/fpsyg.2019.02677 PMC6896901 31849779

[B6] Baldini V. Di Stefano R. Rindi L. V. Ahmed A. O. Koola M. M. Solmi M.. (2023). Association between adverse childhood experiences and suicidal behavior in schizophrenia spectrum disorders: a systematic review and meta-analysis. Psychiat. Res. 329:115488. 10.1016/j.psychres.2023.115488 37769371

[B7] Berking M. Eichler E. Luhmann M. Diedrich A. Hiller W. Rief W. (2019). Affect regulation training reduces symptom severity in depression–A randomized controlled trial. PLoS ONE 14:e0220436. 10.1371/journal.pone.0220436 31465443 PMC6715183

[B8] Bernanke J. A. Stanley B. H. Oquendo M. A. (2017). Toward fine-grained phenotyping of suicidal behavior: The role of suicidal subtypes. Mol. Psychiat. 22, 1080–1081. 10.1038/mp.2017.123 28607457 PMC5785781

[B9] Bhosle S. H. Zanjad N. P. Dake M. D. Godbole H. V. (2015). Deaths due to hanging among adolescents–a 10-year retrospective study. J Forensic Leg Med. 29, 30–33. 10.1016/j.jflm.2014.11.003 25572082

[B10] Blankstein K. R. Lumley C. H. Crawford A. (2007). Perfectionism, hopelessness, and suicide ideation: revisions to diathesis-stress and specific vulnerability models. JRE CBT. 25, 279–319. 10.1007/s10942-007-0053-6

[B11] Boudreaux E. D. Rundensteiner E. Liu F. Wang B. Larkin C. Agu E.. (2021). Applying machine learning approaches to suicide prediction using healthcare data: overview and future directions. Front. Psychiat. 12:707916. 10.3389/fpsyt.2021.707916 34413800 PMC8369059

[B12] Calvete E. Connor-Smith J. K. (2007). Perceived social support, coping, and symptoms of distress in American and Spanish students. Anxiety Stress Coping 19, 47–65. 10.1080/10615800500472963

[B13] Carpenter G. S. J. Carpenter T. P. Kimbrel N. A. Flynn E. J. Pennington M. L. Cammarata C.. (2015). Social support, stress, and suicidal ideation in professional firefighters. Am. J. Health Behav. 39, 191–196. 10.5993/AJHB.39.2.5 25564831

[B14] Carver C. S. (1997). You want to measure coping but your protocol's too long: consider the Brief COPE. Int. J. Behav. Med. 4, 92–100. 10.1207/s15327558ijbm0401_6 16250744

[B15] Cepuch G. Kruszecka-Krówka A. Liber P. Micek A. (2023). Association between suicidal behaviors in adolescence and negative emotions, the level of stress, stress coping strategies and the quality of sleep. Healthcare. 11:306. 10.3390/healthcare11030306 36766881 PMC9914235

[B16] Cheng Q. Wang H. Wei H. Zhang X. Xue T. (2020). Perceived social support from family members and friends and suicidal ideation in Chinese older adults: the buffering hypothesis of resilience. Aging Mental Health 24, 985–990.30835497

[B17] Chervonsky E. Hunt C. (2017). Suppression and expression of emotion in social and interpersonal outcomes: a meta-analysis. Emotion 17:669. 10.1037/emo0000270 28080085

[B18] Chou W. P. Yen C. F. Liu T. L. (2018). Predicting effects of psychological inflexibility/experiential avoidance and stress coping strategies for internet addiction, significant depression, and suicidality in college students: a prospective study. Int. J. Environ. Res. Public Health 15:788. 10.3390/ijerph15040788 29670025 PMC5923830

[B19] Chu H. Yang Y. Zhou J. Wang W. Qiu X. Yang X.. (2021). Social support and suicide risk among Chinese university students: a mental health perspective. Front. Public Health 9:566993. 10.3389/fpubh.2021.566993 33681117 PMC7925394

[B20] Correll C. U. Solmi M. Croatto G. Schneider L. K. Rohani-Montez S. C. Fairley L.. (2022). Mortality in people with schizophrenia: a systematic review and meta-analysis of relative risk and aggravating or attenuating factors. World Psychiat. 21, 248–271. 10.1002/wps.20994 35524619 PMC9077617

[B21] Crispim M. O. Santos C. M. R. D. Frazão I. D. S. Frazão C. M. F. Q. Albuquerque R. C. R. Perrelli J. G. A. (2021). Prevalence of suicidal behavior in young university students: a systematic review with meta-analysis. Rev. Lat. Am. Enfermagem. 29:e3495. 10.1590/1518-8345.5320.3495 34755776 PMC8584877

[B22] Cruz D. Narciso I. Pereira C. R. Sampaio D. (2013), Risk trajectories of self-destructiveness in adolescence: family core influences. J. Child Fam. Stud. 23, 1172–1181. 10.1007/s10826-013-9777-3

[B23] Cutuli D. (2014). Cognitive reappraisal and expressive suppression strategies role in the emotion regulation: an overview on their modulatory effects and neural correlates. Front. Syst. Neurosci. 8:110157. 10.3389/fnsys.2014.00175 25285072 PMC4168764

[B24] Cvetkovski S. Jorm A. F. MacKinnon A. J. (2017). An analysis of the mental health trajectories of university students compared to their community peers using a national longitudinal survey. Stud. High. Educ. 44, 185–200. 10.1080/03075079.2017.1356281

[B25] Donath C. Graessel E. Baier D. Bleich S. Hillemacher T. (2014). Is parenting style a predictor of suicide attempts in a representative sample of adolescents? BMC Pediatr. 14:113. 10.1186/1471-2431-14-113 24766881 PMC4011834

[B26] Favril L. Yu R. Uyar A. Sharpe M. Fazel S. (2022). Risk factors for suicide in adults: systematic review and meta-analysis of psychological autopsy studies. Evid. Based Mental Health 25, 148–155. 10.1136/ebmental-2022-300549 36162975 PMC9685708

[B27] Franz P. J. Kleiman E. M. Nock M. K. (2021). Reappraisal and suppression each moderate the association between stress and suicidal ideation: preliminary evidence from a daily diary study. Cognit. Ther. Res. 1–8. 10.1007/s10608-021-10214-8

[B28] Freudenstein O. Zohar A. Apter A. Shoval G. Weizman A. Zalsman G. (2011). Parental bonding in severely suicidal adolescent inpatients. Eur. Psychiat. 26, 504–507. 10.1016/j.eurpsy.2011.01.006 21398097

[B29] Government of India. (2019). National Suicide Prevention Strategy. Ministry of Health and Family Welfare.

[B30] Gratz K. L. Roemer L. (2004). Multidimensional assessment of emotion regulation and dysregulation: development, factor structure, and initial validation of the difficulties in emotion regulation scale. J. Psychopathol. Behav. Assess. 26, 41–54. 10.1023/B:JOBA.0000007455.08539.94

[B31] Gross J. J. (2014). “Emotion regulation: conceptual and empirical foundations,” in Handbook of Emotion Regulation. 2, ed. J. J. Gross (New York, NY: Guilford), 3–20.

[B32] Gross J. J. John O. P. (2003). Individual differences in two emotion regulation processes: implications for affect, relationships, and well-being. J. Person. Soc. Psychol. 85, 348–362. 10.1037/0022-3514.85.2.348 12916575

[B33] Harmer B. Lee S. Duong T. V. H. Saadabadi A. (2024). Suicidal Ideation. Treasure Island (FL): StatPearls Publishing. 33351435

[B34] Holdaway A. S. Luebbe A. M. Becker S. P. (2018). Rumination in relation to suicide risk, ideation, and attempts: Exacerbation by poor sleep quality? J. Affect. Disord. 236, 6–13. 10.1016/j.jad.2018.04.087 29704657 PMC6047760

[B35] Horwitz A. G. Czyz E. K. Berona J. King C. A. (2018). Prospective associations of coping styles with depression and suicide risk among psychiatric emergency patients. Behav. Ther. 49, 225–236. 10.1016/j.beth.2017.07.010 29530261

[B36] Jadav M. M. (2020). Suicidal tendency among male and female in relation to their type of family. Int. J. Soc. Impact. 5:13. 10.25215/2455/0502013

[B37] Joiner T. (2005). Why People Die by Suicide. Cambridge, MA, US: Harvard University Press.

[B38] Jose S. Angelina J. (2020). Efficacy of Psycho-Spiritual Meaning Intervention (PSMI) on depression and suicide ideation of young adults in Kerala, India. Indian J. Posit. Psychol. 11. 10.15614/ijpp.v11i01.5

[B39] Kar N. (2010). Profile of risk factors associated with suicide attempts: a study from Orissa, India. Indian J. Psychiat. 52:48. 10.4103/0019-5545.58895 20174518 PMC2824981

[B40] Karyotaki E. Cuijpers P. Albor Y. Alonso J. Auerbach R. P. Bantjes J.. (2020). Sources of stress and their associations with mental disorders among college students: results of the World Health Organization World Mental Health Surveys International College Student Initiative. *Front*. Psychol. 11:1759. 10.3389/fpsyg.2020.01759 32849042 PMC7406671

[B41] Khoury B. Sharma M. Rush S. E. Fournier C. (2015). Mindfulness-based stress reduction for healthy individuals: a meta-analysis. J. Psychos. Res. 78, 519–528. 10.1016/j.jpsychores.2015.03.009 25818837

[B42] Lee H. S. Kim S. I. Choi I. Y. Lee K. U. (2008). Prevalence and risk factors associated with suicide ideation and attempts in Korean college students. Psychiat. Invest. 5, 86–93. 10.4306/pi.2008.5.2.86 20046350 PMC2796021

[B43] Liang J. Kolves K. Lew B. de Leo D. Yuan L. Ialib M. A.. (2020). Coping strategies and suicidality: a cross-sectional study from China. Front. Psychiat. 11:129. 10.3389/fpsyt.2020.00129 32231596 PMC7083072

[B44] Miller A. B. Esposito-Smythers C. Leichtweis R. N. (2015). Role of social support in adolescent suicidal ideation and suicide attempts. J. Adolesc. Health 56, 286–292. 10.1016/j.jadohealth.2014.10.265 25561384

[B45] Miola A. Gardea-Reséndez M. Ortiz-Orendain J. Nunez N. A. Ercis M. Coombes B. J.. (2023). Factors associated with suicide attempts in the antecedent illness trajectory of bipolar disorder and schizophrenia. Int. J. Bipolar Disor. 11:38. 10.1186/s40345-023-00318-3 38063942 PMC10709261

[B46] Miotto P. Preti A. (2008). Suicide ideation and social desirability among school-aged young people. J. Adolesc. 31, 519–533. 10.1016/j.adolescence.2007.08.004 17868799

[B47] Nicoară R. D. Nicoară A. M. Cosman D. Coman H. G. (2023). Cognitive-behavioural coping strategies as predictor of suicide risk severity. BRAIN 14, 1–20. 10.18662/brain/14.3/458

[B48] Olfson M. Cosgrove C. M. Altekruse S. F. Wall M. M. Blanco C. (2022). Living alone and suicide risk in the United States, 2008–2019. Am. J. Public Health 112, 1774–1782. 10.2105/AJPH.2022.307080 36383944 PMC9670225

[B49] Ong E. Thompson C. (2019). The importance of coping and emotion regulation in the occurrence of suicidal behavior. Psychol. Rep. 122, 192–1210. 10.1177/0033294118781855 29929434 PMC6628463

[B50] Osman A. Bagge C. L. Gutierrez P. M. Konick L. C. Kopper B. A. Barrios F. X. (2001). The Suicidal Behaviors Questionnaire-Revised (SBQ-R): validation with clinical and nonclinical samples. Assessment 8, 443–454. 10.1177/107319110100800409 11785588

[B51] Owen R. Jones S. H. Dempsey R. C. Gooding P. A. (2021). Directly or indirectly? The role of social support in the psychological pathways underlying suicidal ideation in people with bipolar disorder. Int. J. Environ. Res. Public Health. 19:5286. 10.3390/ijerph19095286 35564679 PMC9099991

[B52] Paashaus L. Forkmann T. Glaesmer H. Juckel G. Rath D. Schönfelder A.. (2019). Do suicide attempters and suicide ideators differ in capability for suicide? Psychiat. Res. 275, 304–309. 10.1016/j.psychres.2019.03.038 30953875

[B53] Pillay J. (2021). Suicidal behaviour among university students: a systematic review. S. Afr. J. Psychol. 51, 54–66. 10.1177/0081246321992177

[B54] Preston E. G. Villarosa-Hurlocker M. C. Raposa E. B. Pearson M. R. Bravo A. J. (2022). Protective Strategies Study Team, Fear of negative evaluation and suicidal ideation among college students: the moderating role of impulsivity-like traits. J. Am. Coll. Health. 71, 396–402. 10.1080/07448481.2021.1891919 33759729 PMC9007699

[B55] Ransing R. Arafat S. Y. Menon V. Kar S. K. (2023). National suicide prevention strategy of India: implementation challenges and the way forward. Lancet Psychiat. 10, 163–165. 10.1016/S2215-0366(23)00027-5 36804065

[B56] Rogier G. Chiorri C. Beomonte Zobel S. Muzi S. Pace C. S. Cheung M. W. L.. (2024). The multifaceted role of emotion regulation in suicidality: Systematic reviews and meta-analytic evidence. Psychol. Bull. 150, 45–81. 10.1037/bul0000415 38376911

[B57] Sachs-Ericsson N. Carr D. Sheffler J. Preston T. J. Kiosses D. Hajcak G. (2021). Cognitive reappraisal and the association between depressive symptoms and perceived social support among older adults. Aging Ment. Health 25, 453–461. 10.1080/13607863.2019.1698516 31876170

[B58] Senapati R. E. Jena S. Parida J. Panda A. Patra P. K. Pati S.. (2024). The patterns, trends and major risk factors of suicide among Indian adolescents–a scoping review. BMC Psychiat. 24, 1–16. 10.1186/s12888-023-05447-8 PMC10775453 38195413

[B59] Shaw R. J. Cullen B. Graham N. Lyall D. M. Mackay D. Okolie C.. (2021). Living alone, loneliness and lack of emotional support as predictors of suicide and self-harm: a nine-year follow up of the UK Biobank cohort. J. Affect. Disord. 279, 316–323. 10.1016/j.jad.2020.10.026 33096330 PMC7758739

[B60] Shi B. (2021). Perceived social support as a moderator of depression and stress in college students. Soc. Behav. Person. 49, 1–9. 10.2224/sbp.9893

[B61] Song H. S. Bae S. M. (2020). The moderating effects of the facets of mindfulness on the relationship between daily life stress and suicidal ideation among Korean college students. Int. J. Mental Health Addict. 20, 136–151. 10.1007/s11469-020-00345-6

[B62] Takizawa R. Danese A. Maughan B. Arseneault L. (2017). Bullying victimization in childhood predicts inflammation and obesity at mid-life: a five-decade birth cohort study. Psychol. Med. 47, 878–887. 10.1017/S0033291715000653 25988703

[B63] Tang F. Qin P. (2015). Influence of personal social network and coping skills on risk for suicidal ideation in Chinese university students. PLoS ONE 10:e0121023. 10.1371/journal.pone.0121023 25803665 PMC4372485

[B64] Tasnim R. Islam M. S. Sujan M. S. H. Sikder M. T. Potenza M. N. (2020). Suicidal ideation among Bangladeshi university students early during the COVID-19 pandemic: Prevalence estimates and correlates. Childr. Youth Serv. Rev. 119:105703. 10.1016/j.childyouth.2020.105703 33204046 PMC7654299

[B65] Thompson R. A. Calkins S. D. (1996). The double-edged sword: Emotional regulation for children at risk. Dev. Psychopathol. 8, 163–182. 10.1017/S0954579400007021

[B66] Turton H. Berry K. Danquah A. Green J. Pratt D. (2022). An investigation of whether emotion regulation mediates the relationship between attachment insecurity and suicidal ideation and behaviour. Clin. Psychol. Psychother. 29, 1587–1598. 10.1002/cpp.2735 35297124 PMC9790629

[B67] Van Orden K. A. Witte T. K. Cukrowicz K. C. Braithwaite S. R. Selby E. A. Joiner T. E. (2010). The interpersonal theory of suicide. Psychol. Rev. 117, 575–600. 10.1037/a0018697 20438238 PMC3130348

[B68] Verma T. (2022). Student suicides in India at a five-year high, majority from Maharashtra: NCRB data. The Indian EXPRESS.

[B69] Vijayakumar L. Kumar M. S. Danabalan M. (2019). Suicide prevention and control in India. Indian J. Psychiat. 61, S221–S225. 10.4103/psychiatry.IndianJPsychiatry_606_19

[B70] Wang M. C. Lightsey O. R. Pietruszka T. Uruk A. C. Wells A. G. (2007). Purpose in life and reasons for living as mediators of the relationship between stress, coping, and suicidal behavior. J Posit. Psychol. 2, 195–204. 10.1080/17439760701228920

[B71] Wastler H. M. Núñez D. (2022). Psychotic experiences, emotion regulation, and suicidal ideation among Chilean adolescents in the general population. Front. Psychiat. 13:983250. 10.3389/fpsyt.2022.983250 36465305 PMC9710630

[B72] Werbart Törnblom A. Sorjonen K. Runeson B. Rydelius P. A. (2021). Life events and coping strategies among young people who died by suicide or sudden violent death. *Front*. Psychiatry. 12:670246. 10.3389/fpsyt.2021.670246 34512410 PMC8429488

[B73] Woodhead E. L. Cronkite R. C. Moos R. H. Timko C. (2014). Coping strategies predictive of adverse outcomes among community adults. J. Clin. Psychol. 70, 1183–1195. 10.1002/jclp.21924 23629952

[B74] World Health Organization. (2020). Suicide is complex. World Health Statistics 2020 visual summary. Available online at: https://www.who.int/news-room/fact-sheets/detail/suicide (accessed August 28, 2023).

[B75] World Health Organization. (2023). Preventing Suicide: a Resource for Media Professionals, 2023 Update. Geneva: World Health Organization.

[B76] Yang K. Petersen K. J. Qualter P. (2022). Undesirable social relations as risk factors for loneliness among 14-year-olds in the UK: findings from the Millennium Cohort Study. Int. J. Behav. Devel. 46, 3–9. 10.1177/0165025420965737

[B77] Yin Y. Tong J. Huang J. Tian B. Chen S. Cui Y.. (2020). Suicidal ideation, suicide attempts, and neurocognitive dysfunctions among patients with first-episode schizophrenia. Suicide Life-Threatening Behav. 50, 1181–1188. 10.1111/sltb.12689 32949038

[B78] Zimet G. D. Dahlem N. W. Zimet S. G. Farley G. K. (1988). The multidimensional scale of perceived social support. J. Person. Assess. 52, 30–41. 10.1207/s15327752jpa5201_2

